# FASTdRNA: a workflow for the analysis of ONT direct RNA sequencing

**DOI:** 10.1093/bioadv/vbad099

**Published:** 2023-07-20

**Authors:** Xiaofeng Chen, Yongqi Liu, Kaiwen Lv, Meiling Wang, Xiaoqin Liu, Bosheng Li

**Affiliations:** Shandong Laboratory of Advanced Agricultural Sciences in Weifang, Peking University Institute of Advanced Agricultural Sciences, Weifang, Shandong 261000, China; National Key Laboratory of Wheat Improvement, Peking University Institute of Advanced Agricultural Sciences, Shandong Laboratory of Advanced Agricultural Sciences in Weifang, Weifang, Shandong 261325, China; Shandong Laboratory of Advanced Agricultural Sciences in Weifang, Peking University Institute of Advanced Agricultural Sciences, Weifang, Shandong 261000, China; National Key Laboratory of Wheat Improvement, Peking University Institute of Advanced Agricultural Sciences, Shandong Laboratory of Advanced Agricultural Sciences in Weifang, Weifang, Shandong 261325, China; Shandong Laboratory of Advanced Agricultural Sciences in Weifang, Peking University Institute of Advanced Agricultural Sciences, Weifang, Shandong 261000, China; National Key Laboratory of Wheat Improvement, Peking University Institute of Advanced Agricultural Sciences, Shandong Laboratory of Advanced Agricultural Sciences in Weifang, Weifang, Shandong 261325, China; Shandong Laboratory of Advanced Agricultural Sciences in Weifang, Peking University Institute of Advanced Agricultural Sciences, Weifang, Shandong 261000, China; National Key Laboratory of Wheat Improvement, Peking University Institute of Advanced Agricultural Sciences, Shandong Laboratory of Advanced Agricultural Sciences in Weifang, Weifang, Shandong 261325, China; Shandong Laboratory of Advanced Agricultural Sciences in Weifang, Peking University Institute of Advanced Agricultural Sciences, Weifang, Shandong 261000, China; National Key Laboratory of Wheat Improvement, Peking University Institute of Advanced Agricultural Sciences, Shandong Laboratory of Advanced Agricultural Sciences in Weifang, Weifang, Shandong 261325, China; Shandong Laboratory of Advanced Agricultural Sciences in Weifang, Peking University Institute of Advanced Agricultural Sciences, Weifang, Shandong 261000, China; National Key Laboratory of Wheat Improvement, Peking University Institute of Advanced Agricultural Sciences, Shandong Laboratory of Advanced Agricultural Sciences in Weifang, Weifang, Shandong 261325, China

## Abstract

**Motivation:**

Direct RNA-seq (dRNA-seq) using Oxford Nanopore Technology (ONT) has revolutionized transcript mapping by offering enhanced precision due to its long-read length. Unlike traditional techniques, dRNA-seq eliminates the need for PCR amplification, reducing the impact of GC bias, and preserving valuable base physical information, such as RNA modification and poly(A) length estimation. However, the rapid advancement of ONT devices has set higher standards for analytical software, resulting in potential challenges of software incompatibility and reduced efficiency.

**Results:**

We present a novel workflow, called FASTdRNA, to manipulate dRNA-seq data efficiently. This workflow comprises two modules: a data preprocessing module and a data analysis module. The preprocessing data module, dRNAmain, encompasses basecalling, mapping, and transcript counting, which are essential for subsequent analyses. The data analysis module consists of a range of downstream analyses that facilitate the estimation of poly(A) length, prediction of RNA modifications, and assessment of alternative splicing events across different conditions with duplication. The FASTdRNA workflow is designed for the Snakemake framework and can be efficiently executed locally or in the cloud. Comparative experiments have demonstrated its superior performance compared to previous methods. This innovative workflow enhances the research capabilities of dRNA-seq data analysis pipelines by optimizing existing processes and expanding the scope of analysis.

**Availability and implementation:**

The workflow is freely available at https://github.com/Tomcxf/FASTdRNA under an MIT license. Detailed install and usage guidance can be found in the GitHub repository.

## 1 Introduction

With the rapid development of sequencing technology, life sciences research has entered a new era. Next-generation sequencing (NGS) has emerged as the dominant approach in recent years due to its lower cost, higher accuracy, and greater data throughput than traditional Sanger sequencing (de Lima *et al.* 2022). Transcriptome analysis using RNA sequencing (RNA-seq) has become a standard technique for identifying differentially expressed genes in specific life cycles, aiding in candidate gene selection (de Lima *et al.* 2022). However, NGS has certain limitations. NGSs relatively short-read lengths make it challenging to process large segments of duplications, placing significant computational and prediction burdens on software and hardware resources. Additionally, NGS involves a PCR amplification step that has two significant drawbacks. Firstly, evidence suggests that standard library preparation procedures utilizing PCR amplification can result in uneven read coverage, particularly across AT- and GC-rich regions, thereby introducing GC bias that can impact sequencing (Huptas *et al.* 2016), genome assembly (Oyola *et al.* 2012, Chen *et al.* 2013), and copy number variation detection (Teo *et al.* 2012). GC bias also has a detrimental effect on the accuracy of transcriptome mapping and subsequent counting stages in RNA-seq. Secondly, RNA-seq requires mRNA to be converted into cDNA, followed by complementary base pairing, erasing the modification information in the mRNA.

As a third-generation sequencing technology, Oxford Nanopore Technologies (ONTs) determines base information by monitoring voltage changes as nucleotides pass through nanopores. In contrast to NGS, ONT sequencing utilizes whole strands, avoiding the need for strand breaking. In 2018, the maximum read length for ONT increased to 2.273 Mb (Payne *et al.* 2019), earning it the designation of long-read sequencing. Despite previous concerns regarding its accuracy, ONT has significantly improved through iterations and upgrades (Wang *et al.* 2021). Importantly, ONT can sequence mRNA directly without PCR amplification, mitigating the issue of GC bias in mRNA results. Moreover, base modification information can be preserved using ONT sequencing (Zhao *et al.* 2019). Adopting direct RNA-seq (dRNA-seq) as a new technology for transcriptome analysis has faced various challenges regarding software applications. Some researchers have resorted to using NGS software to analyze ONT data, which can result in data loss or confusion due to different data formats. Additionally, the rapid development of ONT technology has posed difficulties for traditional analysis pipelines, particularly with the outdated FAST5 file format and inefficient algorithms.

**Figure 1.**
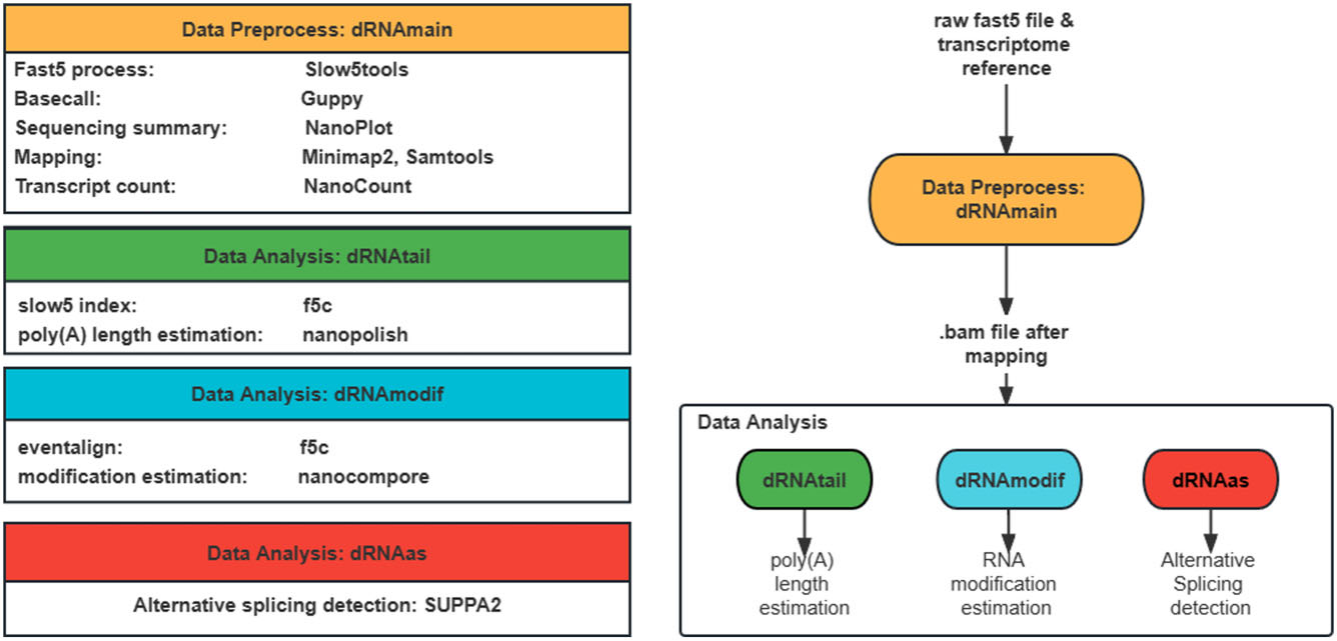
Overview of the FASTdRNA workflow for processing dRNA-seq data from ONT. The flowchart on the right provides an overview of the FASTdRNA workflow. The preprocessing data module involves several steps, including FAST5 processing, basecalling, quality control, sequencing summary, mapping, and transcript counting. The input for this module consists of raw FAST5 files and a transcriptome reference. The output is a BAM file. After obtaining the BAM file from the preprocessing module, the data analysis module can be initiated. This module includes three key analyses: poly(A) length estimation (dRNAtail), RNA modification detection (dRNAmodif), and alternative splicing analysis (dRNAas). The block diagram on the left provides a detailed description of the software used at each workflow step. It illustrates the tools and algorithms employed for FAST5 processing, basecalling, quality control, sequencing summary, mapping, transcript counting, poly(A) length estimation, RNA modification detection, and alternative splicing analysis.

To address these challenges, we propose FASTdRNA, a reliable and efficient pipeline designed explicitly for dRNA-seq analysis. FASTdRNA takes raw FAST5 files and a transcriptome reference as input, and it generates transcript count files for downstream analysis, including differential expression analysis, poly(A) length estimation, RNA modification detection, and alternative splicing information (Fig. 1). Unlike previous dRNA-seq workflows (Cozzuto *et al.* 2020), we have revamped the analysis software and introduced the use of the SLOW5 format to expedite processing. We have also incorporated a new feature for alternative splicing detection. Compared to previous workflows, FASTdRNA significantly improves analysis speed while maintaining accuracy. To achieve this, we have replaced Tombo with more efficient tools for RNA modification estimation, enhancing efficiency and minimizing the impact of the FAST5 file format, resulting in smoother workflow execution. FASTdRNA prioritizes the use of up-to-date software and optimizes the analysis pipeline for dRNA-seq. By following the FASTdRNA workflow, researchers can effectively process dRNA-seq data from ONT, enabling comprehensive analysis of transcriptome features and facilitating downstream investigations.

## 2 Methods

### 2.1 Overview of FASTdRNA

**Figure 2.**
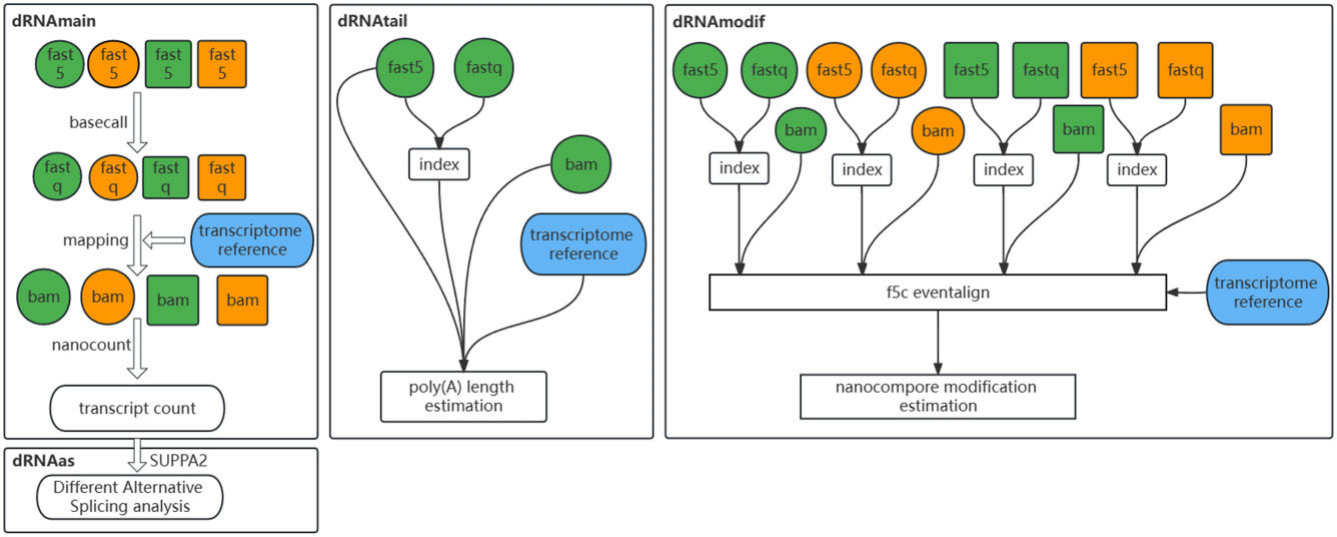
Workflow progression through the execution of dRNAmain, dRNAtail, dRNAmodif, and dRNAas. A dataset with two conditions (indicated by box shape) and two replicates each (indicated by color) were used for test.

Snakemake was selected for the workflow framework due to being Python-based, which makes the pipeline better human readable. This decision considered the bioinformatic defects in computer programming as well as further maintenance and update. Snakemake can be seamlessly scaled to server, cluster, grid, and cloud environments without modifying the workflow definition. For various experiment designs, we separated the whole pipeline into two modules: the data preprocessing module, named dRNAmain, and the data analysis module containing dRNAtail, dRNAmodif, and dRNAas (Fig. 2). Procedure files are retained, as well as the basic visualization, we supply so that researchers will be able to access more details if necessary.

1. dRNAmain: this initial step is crucial for the subsequent three steps. Fast5 files are converted to FASTQ files, mapped to the transcriptome reference to estimate transcript counts.
2. dRNAtail: this step utilizes the FASTQ file, the mapping BAM file, and the transcriptome reference as inputs to estimate poly(A) length using the Nanopolish tool.
3. dRNAmodif: this step aims to detect RNA modifications and requires the BAM file generated by dRNAmain, which includes group and replication information.
4. dRNAas: this step detects alternative splicing events and generates information on AS variations between different conditions or specific controls using annotation and expression files as input.

By executing the dRNAmain, dRNAtail, dRNAmodif, and dRNAas modules of the FASTdRNA pipeline, researchers can effectively process dRNA-seq data, estimate poly(A) length, detect RNA modifications, and analyze alternative splicing events, allowing for a comprehensive exploration of transcriptome features in various experimental conditions.

### 2.2 Data preprocessing: dRNAmain

The dRNAmain step of the FASTdRNA pipeline encompasses two main components: basecall and mapping. Other fundamental data processing steps will be discussed in subsequent sections.

- Basecall: initially, SLOW5tools (Gamaarachchi *et al.* 2022) is utilized to convert FAST5 files to SLOW5 format, a novel format for storing signal data generated by ONT devices. SLOW5 files are smaller and enable more efficient extraction of physical information from raw sequencing data. SLOW5 can be encoded in a human-readable ASCII format or a more efficient binary format called BLOW5. In the FASTdRNA pipeline, the BLOW5 format is selected for downstream analysis. It is essential to distinguish between two types of FAST5 formats: single-FAST5 and multi-FAST5. Single-FAST5 is the previous version of FAST5 files generated by ONT devices. Its notable characteristic is that the output FAST5 directory contains subfolders rather than a list of individual FAST5 files. While moving the FAST5 files from each subfolder into a single folder would enable the smooth execution of Guppy, it can lead to confusion when indexing sequencing information from FASTQ to raw FAST5 using Nanopolish. This issue can be addressed within SLOW5tools, which automatically converts single-FAST5 files into multiple FAST5 files while converting SLOW5 back to FAST5. Furthermore, Guppy, the current official software for basecalling, does not support direct basecalling from SLOW5 format. To overcome this limitation, SLOW5 files are converted back into FAST5 format. The exported FAST5 file releases large quantities of “unaccounted” space compared to the original fast5 file. Bonito, an alternative software to Guppy, can process SLOW5 datasets. However, Bonito currently only supports basecalling for DNA datasets, and future updates may enable RNA basecalling.
- Mapping: the Minimap2 aligner (Li 2018, 2021) maps the FASTQ files to a reference transcriptome. The SAM file from Minimap2 is processed using Samtools (Li *et al.* 2009). Finally, transcript counts are estimated using NanoCount (Gleeson *et al.* 2022), a tool specifically designed for dRNA-seq analysis. Additional scripts are used to generate mapping statistics and facilitate differential expression analysis. The files generated for transcript count estimation are preserved for further research.

If the organism being sequenced has a pre-existing transcriptome reference, it can be directly used for mapping with Minimap2. However, if a transcriptome reference is not available, Gffread (Pertea and Pertea 2020) is employed to merge the genome reference and RNA annotation files into a unified transcriptome reference. To avoid mapping errors, it is crucial to ensure that the merged genome reference and RNA annotation files are derived from the same dataset.

### 2.3 Data analysis: dRNAtail, dRNAmodif, and dRNAas

- dRNAtail: poly(A) length estimation was performed using Nanopolish. To improve efficiency, the SLOW5 format was chosen as the input, considering the time-consuming nature of extracting physical base information. The output file provides the estimated poly(A) length and its precise physical position.
- dRNAmodif: traditionally, the software of choice for detecting RNA modifications from dRNA-seq data has been Tombo. However, Tombo only accepts single-FAST5 files as input, requiring converting multi-FAST5 files to single-FAST5 format. Moreover, Tombo is no longer actively maintained. As an alternative, nanocompore (Leger *et al.* 2021) was selected for RNA modification detection, which requires the availability of a control group. We provide a pipeline utilizing Tombo for RNA modification detection for datasets without a control group, considering its advantage of analyzing data without a control group.
- dRNAas: SUPPA2 (Trincado *et al.* 2018) was modified to detect alternative splicing events. The analysis focuses on local alternative splicing events and different transcript isoforms. The output results include a “.psivec” file generated by deltaPSI (Lin and Krainer 2019), a novel counting strategy developed by SUPPA, which expresses the enrichment of specific transcripts or events. Researchers can identify alterations in transcripts or local alternative splicing events during the designed biological process. Additionally, two official pipeline visualizations are provided. One is a volcano-like plot illustrating the variation in transcript counts and confidence levels. The other is the result of cluster analysis, which groups local alternative splicing events with similar changes in PSI values across conditions into the same cluster.

By utilizing the dRNAtail, dRNAmodif, and dRNAas modules of the FASTdRNA pipeline, researchers can accurately estimate poly(A) length, detect RNA modifications, and analyze alternative splicing events gaining valuable insights into transcriptome dynamics and regulation.

### 2.4 CPU and GPU computing resources

The performance of FASTdRNA was evaluated using both local computing resources and Amazon Elastic Compute Cloud (Amazon EC2). For local computing, the CPU utilized was Intel(R) Xeon(R) Gold 6230R (2.10 GHz), while GPU computing utilized the NVIDIA A100-SXM4-40GB. In the case of Amazon EC2, the CPU computing resources utilized the AMD EPYC 7571 to run the entire pipeline.

## 3 Results

### 3.1 Test for workflow: analysis of *Triticum aestivum* L. mutants

**Figure 3.**
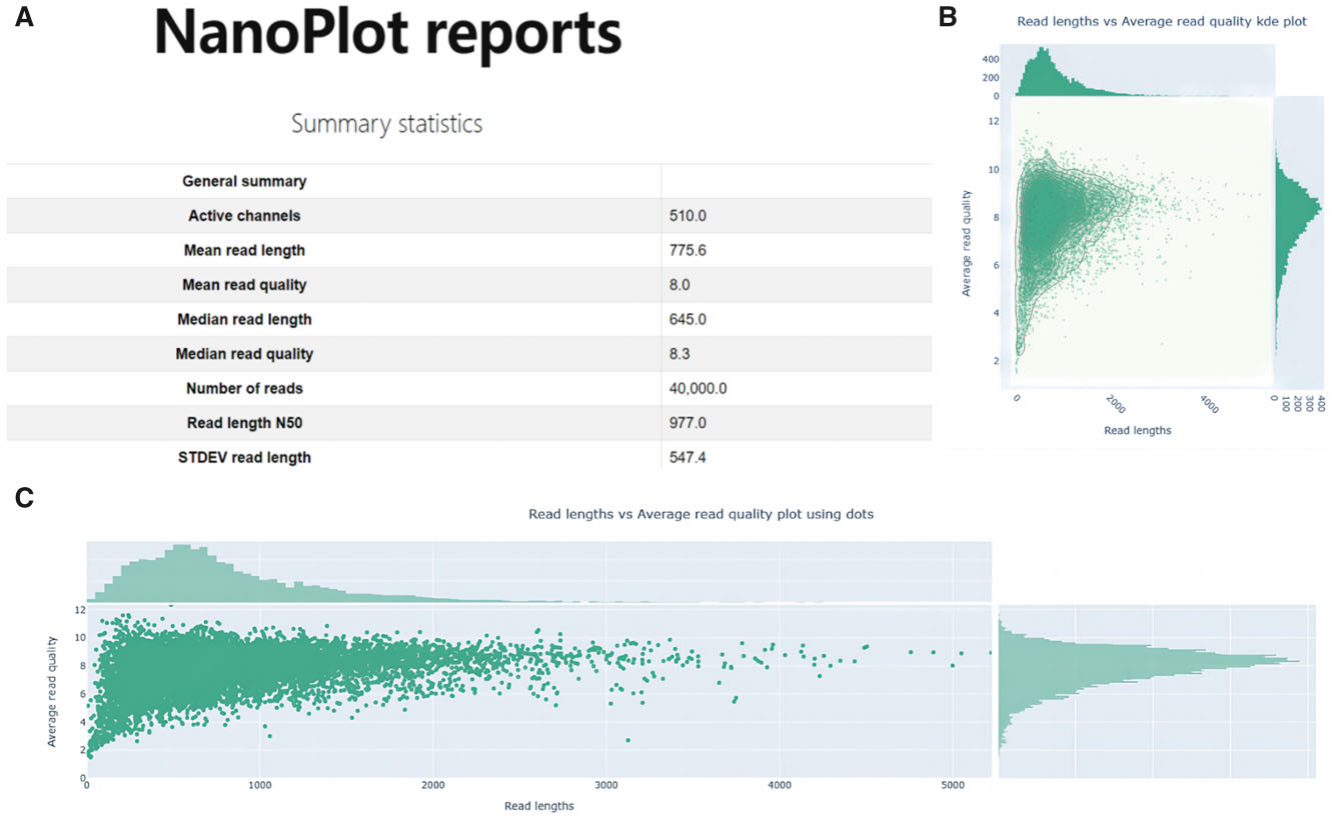
Snapshots of the summary report generated by NanoPlot. (A) Main menu and overview of the summary report, including sequencing and alignment status. (B and C) Partial plots from the summary report for improved visualization of sequencing quality.

**Figure 4.**
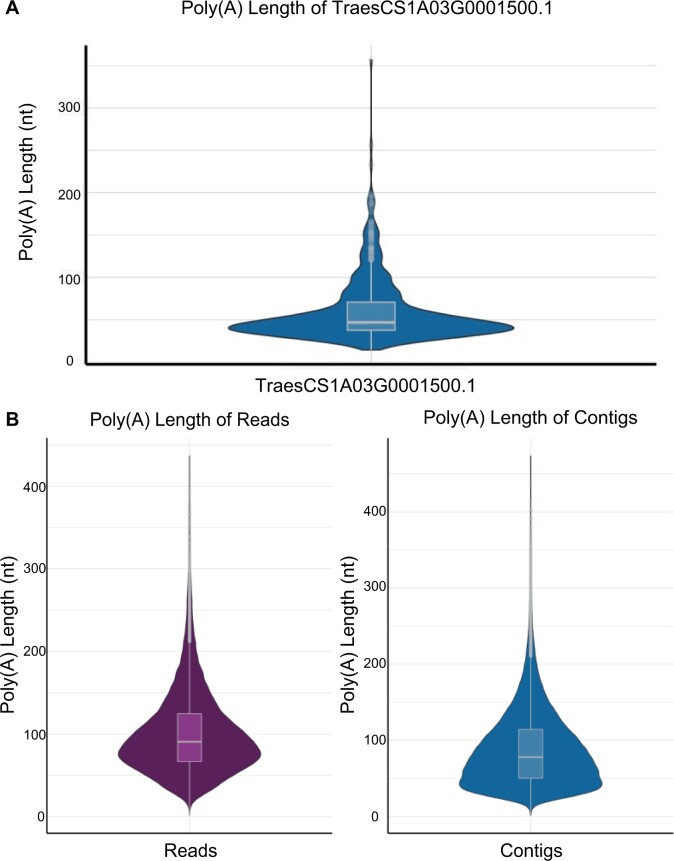
Example outputs of dRNAtail. (A) Visualization of the poly(A) tail length of a specific transcript of interest (e.g. TraesCS4A03G0460700.1 for WT-1). (B) The overview distribution of poly(A) length for reads or contigs is automatically generated.

**Figure 5.**
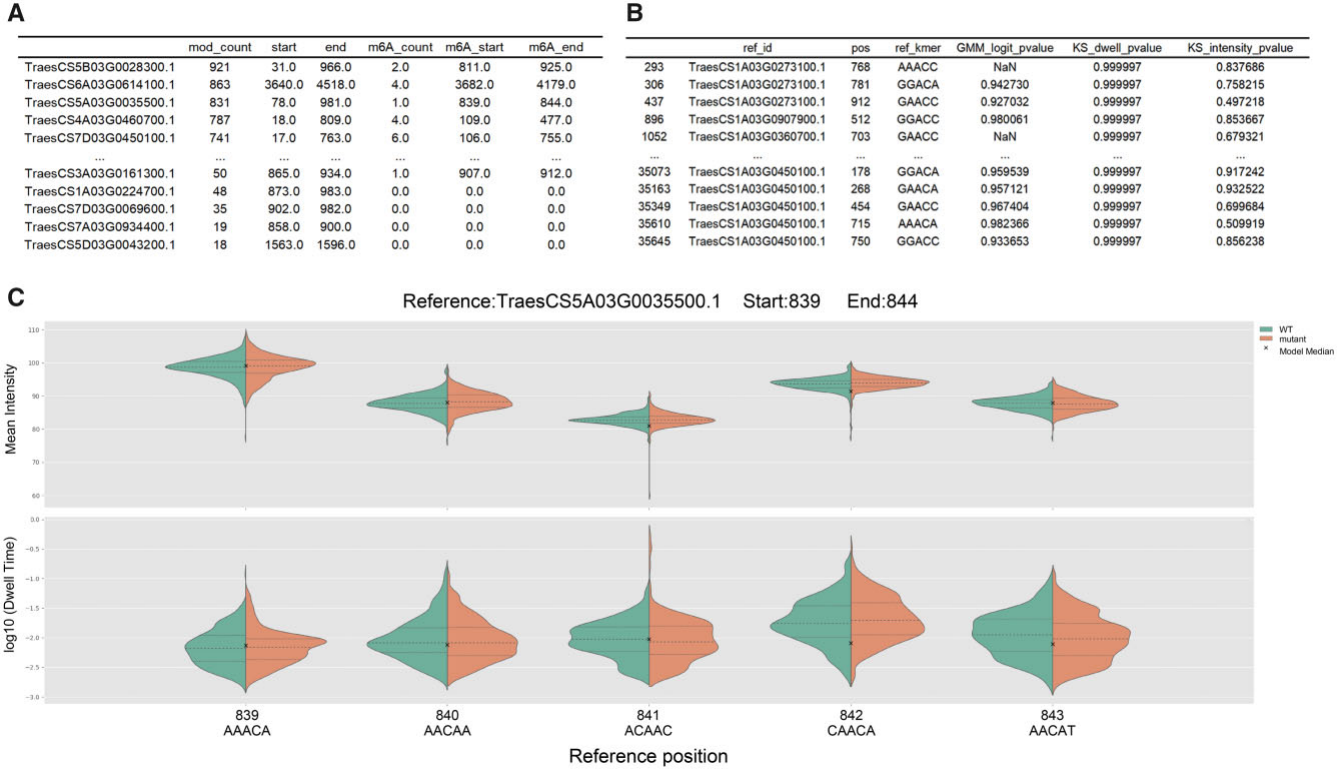
Output of FASTdRNA:dRNAmodif for WT-1. (A) Summary of potential methylation modification sites in each transcript, including m6A modification sites, their counts, and relative positions within the corresponding transcripts. (B) Detailed information on m6A modifications extracted from the raw output. (C) Users can generate Nanocompore’s official visualization figure based on the summary file provided.

To verify the performance of FASTdRNA in an experimental setting, we collected two types of *Triticum aestivum* L. samples, namely wild-type (WT) and mutant, each with two biological replicates. The analysis was conducted using eight cores and 16 threads in CPU mode. In GPU mode, all eight cores share the same GPU for accelerated computing. Table 1 demonstrates the successful execution of FASTdRNA in an actual experiment. Within FASTdRNA, NanoPlot (version 1.41.0) was utilized to provide a summary report displaying sequencing quality and alignment information (Fig. 3). In the dRNAtail module, an overview violin plot was employed to illustrate the poly(A) length distribution of reads and contigs. Additionally, an interface was provided to display the poly(A) tail length of user-selected transcripts of interest (Fig. 4). For the dRNAmodif module, nanocompore (version 1.0.4) was used to predict potential methylation sites on RNA. A summary file containing comprehensive information on all modifications, including m6A methylation, was generated. In addition, users had the option to select transcripts of interest for visualization of all methylation or specifically m6A modifications (Fig. 5). In the dRNAas module, SUPPA2 was employed to search for alternative splicing events using annotation files and predict the probability of each event based on the sequencing data. The output provided by SUPPA2 served as the final report, as it already contained intuitive and comprehensive information, including various alternative splicing types within each transcript and the specific splicing position information for each splicing form. The FASTdRNA's operational logs are included in the ‘Test' subfolder of the Supplementary File.

**Table 1.** Running time of each component of FASTdRNA on four datasets.

	WT-1	WT-2	Mutant 1	Mutant 2
Raw fast5 file	9.9G	9.3G	6.9G	7.8G
Number of reads	1 185 212	1 375 897	966 460	869 863
dRNAmain				
CPU mode	3 h 10 min 55 s	2 h 54 min 38 s	2 h 1 min 50 s	2 h 22 min 56 s
GPU mode	10 min 16 s	9 min 50 s	12 min 14 s	13 min 57 s
dRNAtail				
	1 h 5 min 27 s	1 h 3 min 12 s	38 min 7 s	45 min 55 s
dRNAmodif				
modif1	1 h 36 min 14 s	1 h 30 min 28 s	43 min 29 s	50 min 12 s
modif2	7 min 49 s			
dRNAas				
	30 s			

### 3.2 Comparison between FASTdRNA and MasterOfPores

MasterOfPores is another workflow for analyzing dRNA-seq data, written in Nextflow. However, due to issues with the code of the latest MasterOfPores (MOP2) version, it could not successfully run the four example datasets provided by FASTdRNA, as mentioned below. Therefore, for this comparison, we used the example datasets FAL and FAK from MOP2’s test data and compared the runtime of MOP2 and FASTdRNA. In this comparison, the MOP2 modules mop_preprocess, mop_tail, and mop_mod correspond to the FASTdRNA modules dRNAmain, dRNAtail, and dRNAmodif, respectively. Both workflows were executed using 36 cores and 64 threads, with mop_preprocess and dRNAmain utilizing a dedicated graphics card for computation. In the modification module, we chose the nanocompore algorithms in MOP2, which share the same method as FASTdRNA. Table 2 presents the running time of each pipeline.

**Table 2.** Comparison of running time between MOP2 and FASTdRNA.^a^

	FAK	FAL
Raw fast5 size	48G	117G
Number of reads	227 693	573 309
MOP2_mop_preprocess		
	1 h 39 min 14 s	1 h 58 min 39 s
FdR_dRNAmain		
	19 min 3 s	35 min 35 s
MOP2_mop_tail		
	4 h 36 min 13 s	19 h 46 min 47 s
FdR_dRNAtail		
	5 min 50 s	11 min 12 s
MOP2_mop_mod (nanocompore module)		
	21 h 44 min 33 s	
FdR_dRNAmodif		
modif1	3 h 54 min 41 s	7 h 57 min 25 s
modif2	19 min 31 s	
sum	12 h 11 min 37 s	

a The results indicate that FASTdRNA significantly speeds up the analysis process. Here, FdR represents FASTdRNA.

From the recorded times, it is evident that FASTdRNA has improved analysis efficiency by 2–10 times, partly due to the adoption of the SLOW5 data format in the analysis pipeline. Additionally, while Nextflow is known for its intelligent resource allocation, it is not as flexible in allocating computing resources as Snakemake. Moreover, the more complex code structure of Nextflow makes debugging MOP2 more challenging compared to FASTdRNA. Due to the characteristics of Nextflow, MOP2 requires a decision on whether to use Docker or Singularity as containers. Docker, although widely used, requires high permissions, and improper operations may have serious consequences, such as system crashes.

Compared to MOP2, FASTdRNA provides a more user-friendly visualized report, including a poly(A) length summary for each transcript and potential methylation sites. Additionally, FASTdRNA includes the dRNAas module to detect various alternative splicing events and calculate their probabilities, further expanding the analytical capabilities of the pipeline. The operational logs of the two pipelines are stored in the ‘Compare' subfolder of the Supplementary File.

### 3.3 Running FASTdRNA workflow on AWS EC2

Bioinformatics, a powerful tool for extensive data analysis, often requires advanced computer hardware due to the computational demands of processing large datasets. For ONT data analysis, tasks, such as FAST5 file management, including basecalling, mapping, and modification detection, often exceed the capabilities of personal computers. As a result, sufficient computing resources play a crucial role in determining the efficiency of analysis, particularly for basecalling. To address the limitations of computing resources, we propose utilizing Amazon Web Services Elastic Compute Cloud (AWS EC2). Amazon Web Services (AWS) offers a comprehensive range of global computing, storage, database, analysis, application, and deployment services. It provides several advantages for bioinformatics analysis. Firstly, it offers cost savings in the early stages by replacing upfront hardware investment with a monthly rental model based on actual resource usage. Secondly, AWS allows users to customize their resources according to their specific requirements, enabling scalability, resource utilization improvements, and flexible resource allocation.

Amazon EC2 is a service within AWS that provides scalable computing resources in the cloud. It eliminates the need for upfront hardware investment and allows users to quickly develop and deploy applications, launch virtual servers, configure security and network settings, and manage storage resources based on their needs. Here, we used the first 10 fast5 files of each four samples used in Section 3.1 as datasets and ran it on an Amazon EC2 instance to test its performance, which shows in Table 3. The performance testing of the FASTdRNA pipeline in AWS EC2 environments demonstrated that, when the necessary software and environment configurations are correctly set up, it can operate smoothly and successfully, resembling the experience of working on a local workstation. The operational logs of the FASTdRNA running on Amazon EC2 are stored in the ‘Amazon' subfolder of the Supplementary File.

**Table 3.** Running time of each component of FASTdRNA on Amazon EC2 instance.

	WT-1	WT-2	Mutant 1	Mutant 2
Raw fast5 file	1.7G	1.6G	1.2G	1.5G
Number of reads	222 529	246 966	174 359	173 183
dRNAmain				
	1 h 3 min 23 s	57 min 19 s	38 min 35 s	51 min 56 s
dRNAtail				
	14 min 36 s	13 min 58 s	8 min 33 s	10 min 49 s
dRNAmodif				
modif1	45 min 43 s	42 min 36 s	22 min 13 s	31 min 40 s
modif2	33 s			
dRNAas	4 min 17 s			

### 3.4 SLOW5 accelerates the pipeline and resolves FAST5 format issues

SLOW5 format was initially adopted to enhance the speed of processing raw FAST5 files. Although Guppy does not support basecalling directly from SLOW5 files, converting them back to FAST5 format improved the speed of basecalling compared to using raw FAST5 files. According to the research by the author of slow5tools, there is no data loss during the conversion process when converting from FAST5 -> BLOW5 -> FAST5, and the integrity of the original data is preserved. This suggests that numerous FAST5-based analyses can benefit from this new technique for accelerated calculations.

Furthermore, SLOW5tools automatically converts from single-FAST5 to multi-FAST5 format, simplifying the basecalling process within the workflow. For experiments requiring Tombo for modification event detection, which requires single-FAST5 files, it is highly recommended to use the “multi_to_single_FAST5” function from the “ont_FAST5_api” package to convert the multi-FAST5 files exported by SLOW5tools into single-FAST5 format. This approach ensures compatibility and avoids issues from using raw data directly as input.

## 4 Discussion

With the rapid development of sequencing technology, NGS has become a widely used method for RNA-seq due to its cost-effectiveness and high throughput. However, NGS has limitations, such as short-read length, GC bias introduced by PCR amplification, and loss of RNA structural information during reverse transcription. In contrast, ONT offers a new approach to RNA-seq by directly sequencing RNA strands without PCR amplification, preserving physical RNA information through electrical signal fluctuations, and providing long-read lengths that improve mapping and assembly accuracy.

In this study, we developed a new workflow called FASTdRNA to process dRNA-seq data faster and more robustly. The workflow encompasses basecalling, mapping, transcript count estimation, polyA length estimation, RNA modification detection, alternative splicing detection, and basic visualization capabilities. To overcome the challenges associated with different types of FAST5 formats and to improve efficiency, we utilized SLOW5 files as a substitute for FAST5 files while still utilizing the raw FAST5 files for specific commands. This approach resolves software compatibility issues caused by different FAST5 formats. In addition, the continuous advancements in sequencing technology and computer hardware have accelerated the iteration of bioinformatics algorithms, leading to higher efficiency and accuracy. As a result, each pipeline component is expected to benefit from ongoing improvements and optimizations.

SLOW5, as a new format for storing ONT sequencing raw data, contributes to accelerating the analysis process. Using FAST5 files, the tool that generates SLOW5 files can also generate raw data in ASCII format, improving efficiency and enabling raw FAST5 trimming. Tornado, a tool capable of processing SLOW5 files, provides basecalling functionality, although RNA basecalling is not currently supported. We anticipate that integrating basecalling and mapping in Tornado’s RNA basecalling module, when available, will further enhance the speed of the workflow. A novel method for detecting base modifications, called Remora, has been proposed. These advancements inspire us to pursue more efficient and integrated developments.

In our workflow, Nanocompore was chosen as the tool for RNA modification detection instead of Tombo. While Tombo was excluded from our workflow due to its outdated version and lack of maintenance, it still holds value in scenarios with no duplication or control group. In addition, the publisher has announced the release of Tombo2 at the Nanopore London Calling event, which will further expand the field of using machine learning to study RNA modifications in single-sample contexts.

## 5 Installation and user guidance

### 5.1 Installation

It is recommended to install FASTdRNA through Conda, a widespread software manager. One of its channels, bioconda, can manage hundreds of bioinformatic software and solve dependency issues.

In FASTdRNA, we provide a YAML file to help users generate a software environment and install related dependencies with just one click. Download from our Github mainpage, just type $conda env create -f environment.yml.

### 5.2 Usage

Before running the workflow, certain datasets are required: raw FAST5 data, transcriptome reference directly downloaded from the public database or manually generated. To run dRNAas, an annotation file in .gtf format is required for extracting local AS event.

It is necessary to modify the config file to inform FASTdRNA the precise location of the document needed.

With the preparation above, the pipeline can be run through $Snakemake -s dRNAmain.py/dRNAtail.py/dRNAmodif.py/dRNAas.py.

## Supplementary Material

vbad099_Supplementary_Data

## Data Availability

The raw sequence data reported in this article have been deposited in the Genome Sequence Archive (Chen *et al.* 2021) in National Genomics Data Center (CNCB-NGDC Members and Partners 2022), China National Center for Bioinformation/Beijing Institute of Genomics, Chinese Academy of Sciences (PRJCA017047) that are publicly accessible at https://ngdc.cncb.ac.cn/gsa.
